# An External Urethrotomy

**Published:** 1911-06

**Authors:** Frank P. Norman

**Affiliations:** Greenville, Ga.


### "AN EXTERNAL URETHROTOMY."

FRANK P. NORMAN, M.D., GREENVILLE, GA.

Patient G. V., colored; age 32, unmarred; occupation, farmer. In July, 1909, had right thigh at lower third for chronic sepsis and necrosis of his leg from knee down, by the writer.

As an indication of his personal equation, I might add that he made an absolutely uneventful recovery, never requiring a single dose of anything succeeding the operation. He was out of the house on crutches within eleven days.

As a prelude to my present account, I would like to state that in the interim, patient contracted gonorrhea, but readily relieved same by injections of solution of "blue stone," according to his account.

On April 3, this year, he came into my office about eleven o'clock a. m., suffering great pain. He had not voided a drop of urine since early the preceding afternoon. He gave no history of chill, temperature was normal and pulse slightly accelerated. Physical examination showed bladder distended almost to umbilicus.

Under proper technique I attempted to pass successively through his urethra a soft rubber catheter and dilating bongie, but neither would pass farther than about the base of the bladder. Unfortunately, I did not have, nor could I procure at the time, and filiforms.

With such results after my efforts and with patient apparently in great agony, I desisted from further manipulation.

I instructed him to return to his home immediately, which was about seven miles from Greenville: after he arrived to take an ounce of salts, and to sit naked in a tub of water as hot as he could well stand it, hoping by these two measures to induce elimination of a kind until I could relieve him.

I arrived at his home several hours afterward and found him sitting in his tub of hot water, still in great pain, and not having voided any urine.

To my mind there could be no clearer cut indications than these for an external urethrotomy. Patient still had good circulation, with no temperature, but with no prospects of a bladder evacuation through the urethra.

Patient himself now shaved his pubes, genitalia and perineum. Having myself scrubbed up, sterilized my instruments and arranged sponges, dressings, etc., on a table convenient, I had patient placed across the bed with buttocks in a Kelly pad. I anaesthetized him and then surrendered the job to his father. With my hand and field as near sterile as possible, and with the patient's two brothers holding a thigh respectivety, I passed a No. 8 sound down to the stricture. One of my assistants now bulged this against the perineum, and with my scapel I readily cut down and through the stricture, when from two to four pints of offensive urine poured from the bladder.

With a fountain syringe and a catheter, I irrigated the urethra, bladder and wound with a hot normal saline. I left in situ a catheter in the urethra, and dressed the perineal wound with loose iodoform gauze and a "T" bandage.

I afterwards made several trips to see the patient, removed the catheter on the eighth day, and since the fourteenth day succeeding the operation, he has been visiting my office to have sounds passed. The perineal wound is about closed, his urethra receives a 22 sound and he says he feels very well.

This case has been of a great deal of interest to me and I report it to show that we can sometimes accomplish what appeared to be preposterous before we were brought face to face with it.

---

As part of a hernioplasty it is always worth while to reduce by sutures the hiatus in the transversalis fascia whenever this can be conveniently done.—American Journal of Surgery.

